# Aqua­chlorido­(2-{[6-(di­methyl­amino)­pyrimidin-4-yl]sulfan­yl}pyrimidine-4,6-di­amine)­copper(II) chloride hydrate

**DOI:** 10.1107/S205698901701338X

**Published:** 2017-09-25

**Authors:** Tristen E. Moyaert, Christina Paul, Weibin Chen, Amy A. Sarjeant, Louise N. Dawe

**Affiliations:** a Department of Chemistry and Biochemistry, Faculty of Science, Wilfrid Laurier University, 75 University Ave. W., Waterloo, ON, N2L 3C5, Canada; b Cambridge Crystallographic Data Centre, Center for Integrative Proteomics Research, 174 Frelinghuysen Road, Piscataway, NJ 08854 USA

**Keywords:** crystal structure, copper(II), non-symmetric sulfanyl ligand, disorder

## Abstract

A copper(II) complex of the non-symmetric bidentate ligand 2-{[6-(di­methyl­amino)­pyrimidin-4-yl]sulfan­yl}pyrimidine-4,6-di­amine (*L*1) is reported. The single-crystal X-ray structure of aqua­[aqua/chlorido­(0.49/0.51)](2-{[6-(di­methyl­amino)­pyrimidin-4-yl]sulfan­yl}pyrimidine-4,6-di­amine)­copper(II) 0.49-chloride 1.51-hydrate, [CuCl_1.51_(C_10_H_13_N_7_S)(H_2_O)_1.49_]Cl_0.49_·1.51H_2_O or [(*L*1)Cl_1.51_(H_2_O)_1.49_Cu]0.49Cl·1.51H_2_O, exhibits distorted square-pyramidal geometry around the metal centre, with disorder in the axial position, occupied by chloride or water. The six-membered metal–chelate ring is in a boat conformation, and short inter­molecular S⋯S inter­actions are observed. In addition to its capacity for bidentate metal coordination, the ligand has the ability to engage in further supra­molecular inter­actions as both a hydrogen-bond donor and acceptor, and multiple inter­actions with lattice solvent water mol­ecules are present in the reported structure.

## Abstract 2

The copper(II) complex of the non-symmetric, bidentate ligand 2-{[6-(di­methyl­amino)­pyrimidin-4-yl]sulfan­yl}pyrimidine-4,6-di­amine exhibits distorted square pyramidal geometry around the metal centre, with disorder in the axial position, occupied by chloride or water. The six-membered metal–chelate ring is in a boat-conformation, and short inter­molecular S⋯S inter­actions are observed.

## Chemical context   

**Figure 1:**
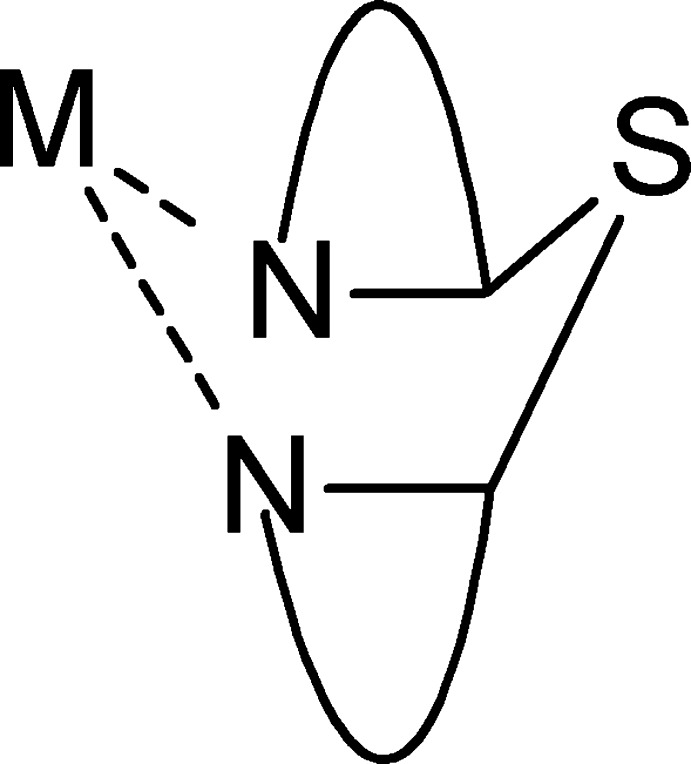
Boat-shaped chelate ring for bidentate coordination of thio-bis-(pyridin-2-yl) or bis-(pyrimidin-2-yl) ligands.

Non-symmetric ligand–metal complexes have been explored for their applications in chiral synthesis (Asay & Morales-Morales, 2015 ▸; Pfaltz & Drury, 2004 ▸), or for their potential to yield new multimetallic topologies which combine homo- and heteroleptic sites into a single mol­ecule (Dawe *et al.*, 2006 ▸). Non-symmetric thio-bis-(pyridin-2-yl) or bis-(pyrimidin-2-yl) ligands are known, and upon bidentate coordination with transition metal cations, these form six-membered chelate rings, which adopt a boat-shaped conformation (Fig. 1 ▸). Some reported transition metal complexes resulting from this class of ligands have been employed as possible alternatives to traditional chemotherapy drugs (Ray *et al.*, 1994 ▸; Mandal *et al.*, 2007 ▸), as a step en route to new thrombin inhibitors (Chung *et al.*, 2003 ▸), and have led to the formation of one Cu^I^ 30-nuclear cluster (Li *et al.*, 2012 ▸).
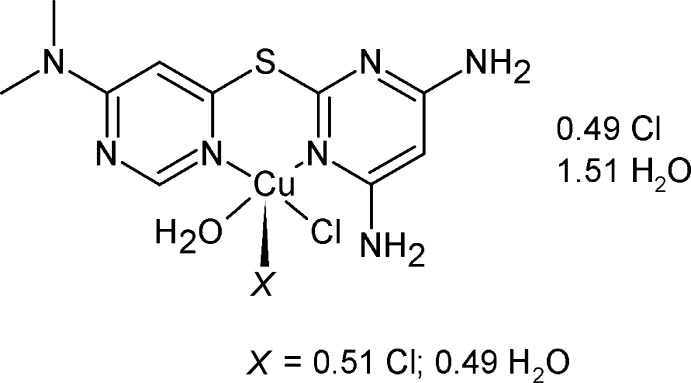


In the inter­est of exploring simultaneous coordination chemistry and anion–ligand affinity *via* hydrogen-bonding inter­actions, the non-symmetric ligand 2-{[6-(di­methyl­amino)pyrimidin-4-yl]sulfan­yl}pyrimidine-4,6-di­amine (C_10_H_13_N_7_S; *L*1), was synthesized, and its metal complex with copper(II) chloride, is reported here. Even upon metal coordination, the ligand can still serve as a hydrogen-bond donor to anions *via* the amine moieties. Alternatively, these free amines could also act as possible anchors for surface attachment, with a view towards future device applications.

## Structural commentary   

**Figure 2:**
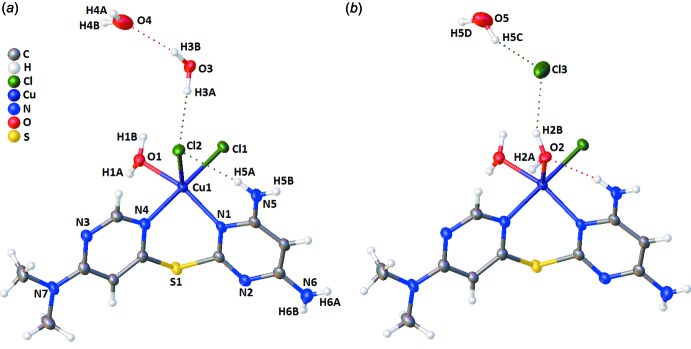
Asymmetric unit for [(C_10_H_13_N_7_S)Cl_1.51_(H_2_O)_1.49_Cu]0.49Cl·1.51H_2_O, with 50% displacement ellipsoids. (*a*) Disordered atoms with 0.51-occupancy; (*b*) disordered atoms with 0.49-occupancy. All atoms in (*a*) and (*b*) are identical, except those labelled in (*b*). Hydrogen bonds are represented by dashed lines.

The title compound crystallizes in the monoclinic space group *P*2_1_/*c* with one bidentate ligand bound to a copper(II) cation (*via* N1 and N4; Fig. 2 ▸). The copper(II) cation is five-coordinate, with the remaining coordination sites occupied by a chloride anion (Cl1), a water mol­ecule (O1), and a disordered site, with either chloride (Cl2; Fig. 2 ▸ *a*) or water (O2; Fig. 2 ▸ *b*) with occupancies of 0.511 (5) and 0.489 (5), respectively. The two largest ligand–metal–ligand bond angles (Table 1 ▸) are N4—Cu1—Cl1 and O1—Cu1—N1 [171.10 (6) and 157.01 (8)°, respectively] giving a τ value of 0.23 (where τ = 0 is ideal square-pyramidal geometry, and τ = 1 is ideal trigonal-bipyramidal geometry; Addison *et al.*, 1984 ▸), indicating that the geometry is distorted square pyramidal. Examination of the bond lengths (Table 1 ▸), is also consistent with the disordered Cl/O as the axial site for this geometry. An intra­molecular hydrogen bond is present between the amine group (*via* N5—H5*A*) and the apical ligand (Fig. 2 ▸; Table 2 ▸). The six-membered chelate ring adopts a boat conformation. The angle between the distorted square plane defined by N1/N4/C4/C5 (r.m.s. deviation from the plane is 0.032 Å) and the flap defined by C4/S1/C5 (θ1) is 34.51 (17)°, while the angle between the square plane and the flap defined by N1/Cu1/N4 (θ2) is 46.93 (14)°. The boat-shaped configuration accommodates the C—S and N—Cu bonds, making up the flaps, which are significantly longer than the C—N bonds in the square plane (Table 1 ▸.)

A simpler, symmetric bidentate ligand, di(pyridin-2-yl)sulf­ide (DPS), has been reported to exhibit a very similar metal coordination environment to the major component reported here, upon reaction with CuCl_2_·H_2_O, to yield [Cu(DPS)(H_2_O)Cl_2_]·H_2_O (Teles *et al.*, 2006 ▸). In this complex, the authors report τ = 0.06, with the square plane formed by the two nitro­gen atoms from DPS, a coordinating water mol­ecule, and one chloride ion (with the second chloride occupying the axial position). Similar to the reported structure here, the six-membered chelate ring adopts a boat conformation, which is characteristic for transition metal complexes with this class of ligands upon bidentate coordination (*vide infra*).

## Supra­molecular features   

**Figure 3:**
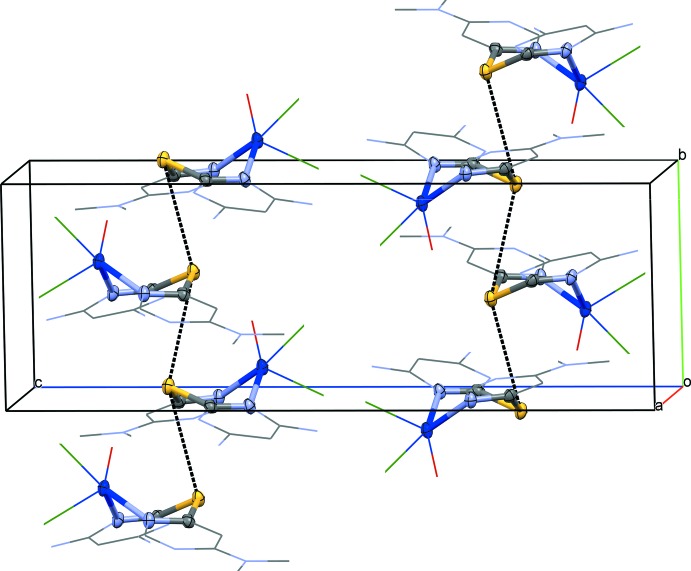
Packed unit cell for [(*L*1)Cl_1.51_(H_2_O)_1.49_Cu]0.49Cl·1.51H_2_O. Only atoms in the major occupancy component are shown. All solvent water mol­ecules and hydrogen atoms have been omitted for clarity.

In the crystal, mol­ecules of the title complex pack in columns, parallel to the crystallographic *b* axis (Fig. 3 ▸), with short S⋯S^i^ inter­molecular distances [3.7327 (3) Å; symmetry code: (i) −*x* + 1, *y* + 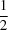, −*z* + 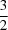]. Note that each chelated ‘boat’ points in the same direction within a column, and the opposite direction is observed in adjacent columns.

## Database survey   

A survey was performed of the Cambridge Structural Database (version 5.38 with May 2017 updates; Groom *et al.*, 2016 ▸), using *ConQuest* (version 1.19; Bruno *et al.*, 2002 ▸), for six-membered transition metal chelate rings resulting from bidentate ligand coordination, where the metal was any transition metal, and the other ring components were N–C–S–C–N. Further, within the ligand, each C—N was required to be part of a six-membered ring, where the remaining four atoms could be any non-metal, and the bond type within the ring was unspecified (allowed to be ‘any’ bond type). This resulted in 74 hits, which were then manually sorted to omit systems where the ligand exhibited anything greater than bidenticity, leaving 68 structures for further analysis using *Mercury* (version 3.9; Macrae *et al.*, 2006 ▸). All of these exhibited boat-shaped puckering of the chelate ring, with mean values for θ1 = 43 (7) and θ2 = 37 (5)°. While the larger angle for the title complex is θ2, both θ1 and θ2 are within two standard deviations of comparable structures from the database.

## Synthesis and crystallization   

**2-{[6-(Di­methyl­amino)­pyrimidin-4-yl]sulfan­yl}pyrimidine-4,6-di­amine (C_10_H_13_N_7_S;** ***L*** **1):** 0.972 g (7.03 mmol) of potassium carbonate and 1.000 g (6.24 mmol) of 4,6-di­amino-2-mercapto­pyrimidine hydrate were combined in 20 mL of di­methyl­formamide, and stirred at 333 K for 20 min, prior to the addition of 0.524 g (3.51 mmol) of 4,6-di­chloro­pyrimidine (see reaction scheme). The resulting cloudy orange solution was refluxed for 24 h. It was then filtered, and the brown filtrate was reduced *in vacuo* to yield 0.387 g (1.47 mmol) of orange solid, after washing with ethanol (42% yield).
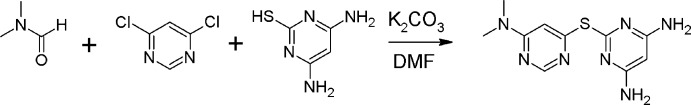


**Aqua­chlorido­(2-{[6-(di­methyl­amino)­pyrimidin-4-yl]sulfan­yl}pyrimidine-4,6-di­amine)­copper(II) chloride hydrate [CuCl_1.51_(C_10_H_13_N_7_S)(H_2_O)_1.49_]Cl_0.49_·1.51H_2_O:** 0.050 g (0.19 mmol) of *L*1 and 0.048 g (0.28 mmol) of CuCl_2_·2H_2_O were separately dissolved in 5 mL of 1:1 methanol/aceto­nitrile. The solution of CuCl_2_ was added dropwise to the solution of *L*1. The resulting cloudy brown solution was stirred vigorously with heating (333 K) for 20 min. This was filtered, yielding 0.007 g of brown, amorphous powder, and a clear green filtrate that was left for slow evaporation. Green, prismatic X-ray quality crystals grew from the filtrate over the course of six weeks. 3.6 mg (0.0080 mmol) of analytically pure crystals were harvested as soon as they formed, though the mother liquor was still highly coloured, accounting for the low (4.2%) yield. These crystals were analyzed *via* small mol­ecule X-ray diffraction, and elemental analysis. Analysis calculated for [(C_10_H_13_N_7_S)CuCl_2_·3H_2_O: C, 26.58; H, 4.24; N, 21.7. Found: C, 26.26; H, 4.13; N, 21.31. Presence of copper confirmed *via* graphite furnace atomic absorption spectroscopy: calculated: 25 µg L^−1^; found: 31.0 ± 0.14 µg L^−1^ (*n* = 8).

## Refinement   

Crystal data, data collection and structure refinement details are summarized in Table 3 ▸. Hydrogen atoms were introduced in calculated positions and refined using a riding model, except those bonded to oxygen or nitro­gen atoms, which were introduced in difference-map positions. N—H hydrogen atoms were refined isotropically, with no restraints. All O—H hydrogen atoms (all associated with water mol­ecules) were refined with *U*_iso_(H) 1.5 times that of the parent atoms and rotating geometry constraints (AFIX 7). Similar distance restraints (SADI, esd 0.02) were applied for all water mol­ecules.

The structure exhibited significant disorder. This included main fragment disorder in the coordination sphere around Cu1. As such, similar distance restraints (SADI, esd 0.02) were applied to the Cu—OH_2_ and Cu—Cl bonds; for each, one O atom (O1) and one Cl atom (Cl1) were fully occupied, while the other (O2 and Cl2) were at partial occupancy, occupying the same coordination site on Cu1, with a sum of their occupancy equal to one. Identical anisotropic displacement parameter (EADP) constraints were applied to Cl2 and O2. Finally, EADP constraints were also applied to a disordered water mol­ecule (O4 and O5), with a sum occupancy of one.

While the structure does exhibit significant disorder, careful consideration was given to ensure that: (i) charge balance was established; (ii) the model was consistent with a reasonable hydrogen-bonding network; and (iii) the next highest residual electron density peak was associated along a C—S bond.

## Supplementary Material

Crystal structure: contains datablock(s) I. DOI: 10.1107/S205698901701338X/zl2715sup1.cif

Structure factors: contains datablock(s) I. DOI: 10.1107/S205698901701338X/zl2715Isup2.hkl

CCDC reference: 1575371

Additional supporting information:  crystallographic information; 3D view; checkCIF report

## Figures and Tables

**Table 1:** Selected geometric parameters (Å, °)

Cu1—Cl1	2.2689 (7)	Cu1—N4	1.996 (2)
Cu1—Cl2	2.5273 (19)	S1—C4	1.777 (2)
Cu1—O1	2.0158 (19)	S1—C5	1.774 (3)
Cu1—O2	2.229 (6)	N1—C4	1.332 (3)
Cu1—N1	2.034 (2)	N4—C5	1.353 (3)
			
Cl1—Cu1—Cl2	92.89 (5)	N1—Cu1—O2	101.55 (18)
O1—Cu1—Cl1	91.94 (6)	N4—Cu1—Cl1	171.10 (6)
O1—Cu1—Cl2	92.69 (7)	N4—Cu1—Cl2	95.20 (8)
O1—Cu1—O2	100.64 (18)	N4—Cu1—O1	83.98 (8)
O1—Cu1—N1	157.01 (8)	N4—Cu1—O2	95.5 (2)
O2—Cu1—Cl1	93.1 (2)	N4—Cu1—N1	87.99 (8)
N1—Cu1—Cl1	92.81 (6)	C5—S1—C4	104.53 (12)
N1—Cu1—Cl2	109.51 (8)		

**Table 2:** Hydrogen-bond geometry (Å, °)

*D*—H⋯*A*	*D*—H	H⋯*A*	*D*⋯*A*	*D*—H⋯*A*
O1—H1*A*⋯N2^i^	0.92 (1)	1.99 (2)	2.897 (3)	170 (3)
O1—H1*B*⋯O4^ii^	0.91 (2)	1.82 (6)	2.71 (6)	167 (4)
O1—H1*B*⋯O5^ii^	0.91 (2)	1.80 (6)	2.69 (6)	162 (4)
O3—H3*A*⋯Cl2	0.92	2.28	3.200 (7)	176
O3—H3*B*⋯O4	0.93	2.17	2.94 (6)	139
O4—H4*A*⋯O3^ii^	0.91	2.25	3.07 (6)	150
O2—H2*B*⋯Cl3	0.91	2.33	3.177 (7)	154
O5—H5*C*⋯Cl3	0.93	2.02	2.83 (6)	145
N5—H5*A*⋯Cl2	0.84 (3)	2.46 (3)	3.295 (3)	172 (3)
N5—H5*A*⋯O2	0.84 (3)	2.07 (3)	2.903 (7)	167 (3)
N5—H5*B*⋯Cl1^iii^	0.79 (3)	2.56 (4)	3.353 (3)	173 (3)
N6—H6*A*⋯Cl3^iii^	0.80 (4)	2.41 (4)	3.187 (4)	165 (3)
N6—H6*A*⋯O3^iii^	0.80 (4)	2.32 (4)	3.094 (8)	163 (3)

Symmetry codes: (i) 
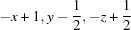
; (ii) 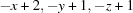; (iii) 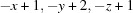.

**Table 3:** Experimental details

Crystal data	
Chemical formula	[CuCl_1.51_(C_10_H_13_N_7_S)(H_2_O)_1.49_]·Cl_0.49_·1.51H_2_O
*M*_r_	451.82
Crystal system, space group	Monoclinic, *P*2_1_/*c*
Temperature (K)	110
*a*, *b*, *c* (Å)	11.42069 (19), 7.23911 (12), 21.6990 (3)
β (°)	103.1543 (16)
*V* (Å^3^)	1746.91 (5)
*Z*	4
Radiation type	Cu *K*α
μ (mm^−1^)	5.94
Crystal size (mm)	0.27 × 0.19 × 0.17

Data collection	
Diffractometer	Bruker APEXII CCD with *CrysAlis PRO* imported SAXI images
Absorption correction	Multi-scan (*CrysAlis PRO*; Rigaku Oxford Diffraction, 2015 ▸)
*T*_min_, *T*_max_	0.034, 0.115
No. of measured, independent and observed [*I* > 2σ(*I*)] reflections	22331, 3000, 2523
*R*_int_	0.051
(sin θ/λ)_max_ (Å^−1^)	0.594

Refinement	
*R*[*F* ^2^ > 2σ(*F* ^2^)], *wR*(*F* ^2^), *S*	0.030, 0.079, 1.04
No. of reflections	3000
No. of parameters	261
No. of restraints	47
H-atom treatment	H atoms treated by a mixture of independent and constrained refinement
Δρ_max_, Δρ_min_ (e Å^−3^)	0.32, −0.32

Computer programs: *APEX2* (Bruker, 2012 ▸), *CrysAlis PRO* (Rigaku OD, 2015 ▸), *SHELXT* (Sheldrick, 2015*a* ▸), *SHELXL2016* (Sheldrick, 2015*b* ▸) and *OLEX2* (Dolomanov *et al.*, 2009 ▸).

## supplementary crystallographic information

### Crystal data

**Table:**

[CuCl_1.51_(C_10_H_13_N_7_S)(H_2_O)_1.49_]·0.49Cl·1.51H_2_O	*F*(000) = 924
*M**_r_* = 451.82	*D*_x_ = 1.718 Mg m^−^^3^
Monoclinic, *P*2_1_/*c*	Cu *K*α radiation, λ = 1.54178 Å
*a* = 11.42069 (19) Å	Cell parameters from 13698 reflections
*b* = 7.23911 (12) Å	θ = 4.0–66.3°
*c* = 21.6990 (3) Å	µ = 5.94 mm^−^^1^
β = 103.1543 (16)°	*T* = 110 K
*V* = 1746.91 (5) Å^3^	Prism, green
*Z* = 4	0.27 × 0.19 × 0.17 mm

### Data collection

**Table:**

Bruker APEXII CCD with CrysAlis PRO imported SAXI images diffractometer	3000 independent reflections
Radiation source: fine-focus sealed X-ray tube, Enhance (Cu) X-ray Source	2523 reflections with *I* > 2σ(*I*)
Graphite monochromator	*R*_int_ = 0.051
ω and φ scans	θ_max_ = 66.4°, θ_min_ = 4.0°
Absorption correction: multi-scan (CrysAlis PRO; Rigaku Oxford Diffraction, 2015)	*h* = −13→13
*T*_min_ = 0.034, *T*_max_ = 0.115	*k* = −8→8
22331 measured reflections	*l* = −25→22

### Refinement

**Table:**

Refinement on *F*^2^	47 restraints
Least-squares matrix: full	Hydrogen site location: mixed
*R*[*F*^2^ > 2σ(*F*^2^)] = 0.030	H atoms treated by a mixture of independent and constrained refinement
*wR*(*F*^2^) = 0.079	*w* = 1/[σ^2^(*F*_o_^2^) + (0.0438*P*)^2^ + 0.8086*P*] where *P* = (*F*_o_^2^ + 2*F*_c_^2^)/3
*S* = 1.04	(Δ/σ)_max_ = 0.001
3000 reflections	Δρ_max_ = 0.32 e Å^−^^3^
261 parameters	Δρ_min_ = −0.32 e Å^−^^3^

### Special details

**Table:**

Geometry. All esds (except the esd in the dihedral angle between two l.s. planes) are estimated using the full covariance matrix. The cell esds are taken into account individually in the estimation of esds in distances, angles and torsion angles; correlations between esds in cell parameters are only used when they are defined by crystal symmetry. An approximate (isotropic) treatment of cell esds is used for estimating esds involving l.s. planes.

### Fractional atomic coordinates and isotropic or equivalent isotropic displacement parameters (Å^2^)

**Table:**

	*x*	*y*	*z*	*U*_iso_*/*U*_eq_	Occ. (<1)
Cu1	0.65886 (3)	0.87641 (5)	0.36726 (2)	0.02056 (12)	
Cl1	0.59471 (5)	0.68609 (9)	0.43587 (3)	0.02412 (16)	
Cl2	0.81961 (17)	1.0193 (3)	0.45364 (9)	0.0250 (6)	0.511 (5)
Cl3	0.9316 (3)	0.7674 (6)	0.55061 (13)	0.0648 (12)	0.489 (5)
S1	0.47778 (6)	0.94220 (9)	0.22968 (3)	0.02244 (16)	
O1	0.77219 (16)	0.6829 (3)	0.34783 (8)	0.0256 (4)	
H1A	0.743 (3)	0.635 (4)	0.3084 (9)	0.051 (11)*	
H1B	0.790 (3)	0.586 (4)	0.3750 (15)	0.062 (12)*	
O3	0.9002 (6)	0.6924 (9)	0.5536 (4)	0.0305 (15)	0.511 (5)
H3A	0.873556	0.784224	0.524170	0.046*	0.511 (5)
H3B	0.982596	0.704284	0.569168	0.046*	0.511 (5)
O4	1.148 (5)	0.571 (8)	0.560 (3)	0.056 (3)	0.511 (5)
H4A	1.164621	0.489914	0.531128	0.084*	0.511 (5)
H4B	1.218311	0.634714	0.578705	0.084*	0.511 (5)
O2	0.7814 (6)	1.0316 (10)	0.4446 (3)	0.0250 (6)	0.489 (5)
H2A	0.816574	1.128467	0.429387	0.038*	0.489 (5)
H2B	0.842833	0.962117	0.467637	0.038*	0.489 (5)
O5	1.141 (5)	0.553 (9)	0.557 (3)	0.056 (3)	0.489 (5)
H5C	1.094042	0.658743	0.549583	0.084*	0.489 (5)
H5D	1.164982	0.487073	0.526489	0.084*	0.489 (5)
N1	0.50737 (19)	1.0333 (3)	0.35292 (9)	0.0214 (5)	
N2	0.31504 (19)	1.0711 (3)	0.28275 (10)	0.0229 (5)	
N3	0.86159 (19)	1.1243 (3)	0.25035 (10)	0.0238 (5)	
N4	0.70468 (19)	1.0147 (3)	0.29669 (9)	0.0213 (5)	
N5	0.5433 (2)	1.1471 (4)	0.45509 (11)	0.0286 (5)	
H5A	0.614 (3)	1.106 (5)	0.4577 (15)	0.034*	
H5B	0.517 (3)	1.185 (5)	0.4834 (16)	0.034*	
N6	0.1509 (2)	1.1695 (4)	0.32017 (14)	0.0321 (6)	
H6A	0.122 (3)	1.202 (5)	0.3488 (17)	0.033 (9)*	
H6B	0.109 (3)	1.134 (5)	0.2867 (17)	0.037 (10)*	
N7	0.8262 (2)	1.1817 (3)	0.14302 (10)	0.0266 (5)	
C1	0.4646 (2)	1.1146 (4)	0.40076 (12)	0.0232 (6)	
C2	0.3434 (2)	1.1595 (4)	0.39165 (12)	0.0250 (6)	
H2	0.312207	1.208633	0.425222	0.030*	
C3	0.2696 (2)	1.1307 (4)	0.33244 (13)	0.0246 (6)	
C4	0.4299 (2)	1.0264 (3)	0.29687 (11)	0.0204 (5)	
C5	0.6270 (2)	1.0255 (3)	0.23952 (11)	0.0205 (5)	
C6	0.6605 (2)	1.0850 (3)	0.18649 (11)	0.0203 (5)	
H6	0.604065	1.095192	0.147050	0.024*	
C7	0.7828 (2)	1.1307 (4)	0.19266 (12)	0.0229 (6)	
C8	0.8173 (2)	1.0684 (4)	0.29833 (12)	0.0235 (6)	
H8	0.871669	1.066177	0.338604	0.028*	
C9	0.7483 (3)	1.1897 (4)	0.07954 (13)	0.0342 (7)	
H9A	0.729605	1.063940	0.063603	0.051*	
H9B	0.789517	1.256260	0.051290	0.051*	
H9C	0.673663	1.254206	0.081154	0.051*	
C10	0.9543 (3)	1.2111 (5)	0.14952 (14)	0.0360 (7)	
H10A	0.984405	1.295187	0.184969	0.054*	
H10B	0.968239	1.265239	0.110432	0.054*	
H10C	0.996500	1.092607	0.157550	0.054*	

### Atomic displacement parameters (Å^2^)

**Table:**

	*U*^11^	*U*^22^	*U*^33^	*U*^12^	*U*^13^	*U*^23^
Cu1	0.0206 (2)	0.0275 (2)	0.0147 (2)	0.00178 (16)	0.00640 (14)	0.00253 (14)
Cl1	0.0240 (3)	0.0325 (3)	0.0171 (3)	0.0027 (3)	0.0072 (2)	0.0045 (2)
Cl2	0.0180 (12)	0.0367 (7)	0.0183 (8)	0.0039 (8)	−0.0002 (7)	0.0004 (5)
Cl3	0.055 (2)	0.110 (3)	0.0285 (11)	0.0397 (19)	0.0078 (11)	−0.0013 (16)
S1	0.0219 (3)	0.0293 (3)	0.0168 (3)	−0.0034 (3)	0.0060 (2)	−0.0018 (2)
O1	0.0215 (10)	0.0351 (11)	0.0208 (10)	0.0028 (8)	0.0059 (8)	0.0012 (8)
O3	0.021 (3)	0.038 (3)	0.035 (3)	0.007 (2)	0.011 (2)	0.012 (2)
O4	0.080 (5)	0.054 (8)	0.034 (4)	0.022 (4)	0.013 (3)	0.009 (4)
O2	0.0180 (12)	0.0367 (7)	0.0183 (8)	0.0039 (8)	−0.0002 (7)	0.0004 (5)
O5	0.080 (5)	0.054 (8)	0.034 (4)	0.022 (4)	0.013 (3)	0.009 (4)
N1	0.0239 (11)	0.0268 (12)	0.0149 (10)	0.0019 (9)	0.0075 (9)	0.0032 (9)
N2	0.0216 (11)	0.0266 (12)	0.0209 (11)	−0.0009 (9)	0.0059 (9)	0.0051 (9)
N3	0.0212 (11)	0.0319 (12)	0.0188 (11)	−0.0002 (10)	0.0055 (9)	0.0019 (9)
N4	0.0212 (11)	0.0276 (12)	0.0156 (10)	0.0006 (9)	0.0051 (8)	−0.0001 (8)
N5	0.0288 (13)	0.0421 (15)	0.0162 (11)	0.0084 (11)	0.0080 (10)	−0.0024 (10)
N6	0.0233 (13)	0.0439 (16)	0.0306 (15)	0.0030 (11)	0.0092 (12)	0.0048 (12)
N7	0.0251 (12)	0.0394 (14)	0.0175 (11)	−0.0014 (10)	0.0092 (9)	0.0033 (9)
C1	0.0276 (14)	0.0244 (14)	0.0199 (13)	0.0024 (11)	0.0100 (11)	0.0068 (10)
C2	0.0285 (15)	0.0282 (14)	0.0213 (13)	0.0046 (11)	0.0117 (11)	0.0054 (10)
C3	0.0253 (14)	0.0234 (14)	0.0277 (14)	0.0016 (11)	0.0113 (11)	0.0101 (11)
C4	0.0236 (14)	0.0200 (13)	0.0191 (12)	−0.0006 (11)	0.0083 (10)	0.0044 (10)
C5	0.0222 (13)	0.0215 (13)	0.0189 (12)	0.0023 (10)	0.0068 (10)	−0.0024 (10)
C6	0.0222 (13)	0.0251 (14)	0.0144 (12)	0.0013 (11)	0.0057 (10)	0.0001 (10)
C7	0.0275 (14)	0.0234 (13)	0.0196 (13)	0.0005 (11)	0.0091 (11)	−0.0029 (10)
C8	0.0238 (14)	0.0292 (14)	0.0174 (12)	0.0023 (11)	0.0047 (10)	0.0013 (10)
C9	0.0341 (16)	0.0502 (18)	0.0193 (14)	−0.0019 (14)	0.0079 (12)	0.0053 (12)
C10	0.0254 (15)	0.057 (2)	0.0291 (15)	−0.0059 (14)	0.0138 (12)	0.0002 (14)

### Geometric parameters (Å, º)

**Table:**

Cu1—Cl1	2.2689 (7)	N4—C5	1.353 (3)
Cu1—Cl2	2.5273 (19)	N4—C8	1.336 (3)
Cu1—O1	2.0158 (19)	N5—H5A	0.84 (3)
Cu1—O2	2.229 (6)	N5—H5B	0.79 (3)
Cu1—N1	2.034 (2)	N5—C1	1.331 (4)
Cu1—N4	1.996 (2)	N6—H6A	0.80 (4)
S1—C4	1.777 (2)	N6—H6B	0.81 (4)
S1—C5	1.774 (3)	N6—C3	1.351 (4)
O1—H1A	0.915 (14)	N7—C7	1.337 (3)
O1—H1B	0.911 (15)	N7—C9	1.461 (3)
O3—H3A	0.9233	N7—C10	1.452 (3)
O3—H3B	0.9290	C1—C2	1.392 (4)
O4—H4A	0.9144	C2—H2	0.9500
O4—H4B	0.9323	C2—C3	1.383 (4)
O2—H2A	0.9063	C5—C6	1.363 (4)
O2—H2B	0.9141	C6—H6	0.9500
O5—H5C	0.9293	C6—C7	1.411 (4)
O5—H5D	0.9107	C8—H8	0.9500
N1—C1	1.376 (3)	C9—H9A	0.9800
N1—C4	1.332 (3)	C9—H9B	0.9800
N2—C3	1.368 (4)	C9—H9C	0.9800
N2—C4	1.318 (3)	C10—H10A	0.9800
N3—C7	1.366 (3)	C10—H10B	0.9800
N3—C8	1.320 (3)	C10—H10C	0.9800
			
Cl1—Cu1—Cl2	92.89 (5)	C7—N7—C10	120.8 (2)
O1—Cu1—Cl1	91.94 (6)	C10—N7—C9	118.0 (2)
O1—Cu1—Cl2	92.69 (7)	N1—C1—C2	120.3 (2)
O1—Cu1—O2	100.64 (18)	N5—C1—N1	117.4 (2)
O1—Cu1—N1	157.01 (8)	N5—C1—C2	122.3 (2)
O2—Cu1—Cl1	93.1 (2)	C1—C2—H2	120.9
N1—Cu1—Cl1	92.81 (6)	C3—C2—C1	118.2 (2)
N1—Cu1—Cl2	109.51 (8)	C3—C2—H2	120.9
N1—Cu1—O2	101.55 (18)	N2—C3—C2	121.3 (2)
N4—Cu1—Cl1	171.10 (6)	N6—C3—N2	117.0 (3)
N4—Cu1—Cl2	95.20 (8)	N6—C3—C2	121.6 (3)
N4—Cu1—O1	83.98 (8)	N1—C4—S1	119.87 (19)
N4—Cu1—O2	95.5 (2)	N2—C4—S1	111.66 (18)
N4—Cu1—N1	87.99 (8)	N2—C4—N1	128.4 (2)
C5—S1—C4	104.53 (12)	N4—C5—S1	120.15 (18)
Cu1—O1—H1A	110 (2)	N4—C5—C6	122.7 (2)
Cu1—O1—H1B	118 (3)	C6—C5—S1	116.92 (19)
H1A—O1—H1B	107 (3)	C5—C6—H6	121.4
H3A—O3—H3B	109.4	C5—C6—C7	117.1 (2)
H4A—O4—H4B	108.7	C7—C6—H6	121.4
Cu1—O2—H2A	111.7	N3—C7—C6	120.7 (2)
Cu1—O2—H2B	114.0	N7—C7—N3	117.4 (2)
H2A—O2—H2B	106.1	N7—C7—C6	122.0 (2)
H5C—O5—H5D	123.9	N3—C8—N4	127.4 (2)
C1—N1—Cu1	124.00 (17)	N3—C8—H8	116.3
C4—N1—Cu1	118.91 (17)	N4—C8—H8	116.3
C4—N1—C1	115.4 (2)	N7—C9—H9A	109.5
C4—N2—C3	115.5 (2)	N7—C9—H9B	109.5
C8—N3—C7	116.2 (2)	N7—C9—H9C	109.5
C5—N4—Cu1	120.02 (17)	H9A—C9—H9B	109.5
C8—N4—Cu1	122.97 (17)	H9A—C9—H9C	109.5
C8—N4—C5	115.7 (2)	H9B—C9—H9C	109.5
H5A—N5—H5B	126 (3)	N7—C10—H10A	109.5
C1—N5—H5A	116 (2)	N7—C10—H10B	109.5
C1—N5—H5B	116 (2)	N7—C10—H10C	109.5
H6A—N6—H6B	122 (4)	H10A—C10—H10B	109.5
C3—N6—H6A	119 (2)	H10A—C10—H10C	109.5
C3—N6—H6B	118 (2)	H10B—C10—H10C	109.5
C7—N7—C9	120.8 (2)		

### Hydrogen-bond geometry (Å, º)

**Table:**

*D*—H···*A*	*D*—H	H···*A*	*D*···*A*	*D*—H···*A*
O1—H1*A*···S1^i^	0.92 (1)	2.83 (3)	3.439 (2)	125 (3)
O1—H1*A*···N2^i^	0.92 (1)	1.99 (2)	2.897 (3)	170 (3)
O1—H1*B*···O4^ii^	0.91 (2)	1.82 (6)	2.71 (6)	167 (4)
O1—H1*B*···O5^ii^	0.91 (2)	1.80 (6)	2.69 (6)	162 (4)
O3—H3*A*···Cl2	0.92	2.28	3.200 (7)	176
O3—H3*B*···O4	0.93	2.17	2.94 (6)	139
O4—H4*A*···Cl1^ii^	0.91	2.97	3.46 (6)	116
O4—H4*A*···O3^ii^	0.91	2.25	3.07 (6)	150
O4—H4*B*···Cl2^iii^	0.93	2.61	3.01 (6)	107
O2—H2*A*···O5^iii^	0.91	2.36	3.13 (6)	144
O2—H2*B*···Cl3	0.91	2.33	3.177 (7)	154
O5—H5*C*···Cl3	0.93	2.02	2.83 (6)	145
O5—H5*C*···O2^iii^	0.93	2.64	3.13 (6)	114
O5—H5*D*···Cl1^ii^	0.91	2.96	3.45 (6)	116
O5—H5*D*···Cl3^ii^	0.91	2.56	3.27 (6)	135
N5—H5*A*···Cl1	0.84 (3)	3.08 (3)	3.430 (3)	108 (2)
N5—H5*A*···Cl2	0.84 (3)	2.46 (3)	3.295 (3)	172 (3)
N5—H5*A*···O2	0.84 (3)	2.07 (3)	2.903 (7)	167 (3)
N5—H5*B*···Cl1^iv^	0.79 (3)	2.56 (4)	3.353 (3)	173 (3)
N6—H6*A*···Cl3^iv^	0.80 (4)	2.41 (4)	3.187 (4)	165 (3)
N6—H6*A*···O3^iv^	0.80 (4)	2.32 (4)	3.094 (8)	163 (3)
N6—H6*B*···N3^v^	0.81 (4)	2.76 (4)	3.323 (3)	128 (3)

Symmetry codes: (i) −*x*+1, *y*−1/2, −*z*+1/2; (ii) −*x*+2, −*y*+1, −*z*+1; (iii) −*x*+2, −*y*+2, −*z*+1; (iv) −*x*+1, −*y*+2, −*z*+1; (v) *x*−1, *y*, *z*.
